# Impact of the Injection Site on Growth Characteristics, Phenotype and Sensitivity towards Cytarabine of Twenty Acute Leukaemia Patient-Derived Xenograft Models

**DOI:** 10.3390/cancers12051349

**Published:** 2020-05-25

**Authors:** Julia Schueler, Gabriele Greve, Dorothée Lenhard, Milena Pantic, Anna Edinger, Eva Oswald, Michael Luebbert

**Affiliations:** 1 Charles River Discovery Research Services Germany GmbH, Am Flughafen 12-14, 79108 Freiburg, Germany; dorothee.lenhard@crl.com (D.L.); anna.edinger@crl.com (A.E.); eva.oswald@crl.com (E.O.); 2 Department of Medicine I, Faculty of Medicine, University of Freiburg, 79106 Freiburg, Germany; gabriele.greve@uniklinik-freiburg.de (G.G.); milena.pantic@uniklinik-freiburg.de (M.P.); michael.luebbert@uniklinik-freiburg.de (M.L.)

**Keywords:** patient-derived xenografts, acute leukaemia, tumour microenvironment, flow cytometry, cytarabine

## Abstract

Rodent models have contributed significantly to the understanding of haematological malignancies. One important model system in this context are patient-derived xenografts (PDX). In the current study, we examined 20 acute leukaemia PDX models for growth behaviour, infiltration in haemopoietic organs and sensitivity towards cytarabine. PDX were injected intratibially (i.t.), intrasplenicaly (i.s.) or subcutaneously (s.c.) into immune compromised mice. For 18/20 models the engraftment capacity was independent of the implantation site. Two models could exclusively be propagated in one or two specific settings. The implantation site did influence tumour growth kinetics as median overall survival differed within one model depending on the injection route. The infiltration pattern was similar in i.t. and i.s. models. In contrast to the s.c. implantation, only one model displayed circulating leukaemic cells outside of the locally growing tumour mass. Cytarabine was active in all four tested models. Nevertheless, the degree of sensitivity was specific for an individual model and implantation site. In summary, all three application routes turned out to be feasible for the propagation of PDX. Nevertheless, the distinct differences between the settings highlight the need for well characterized platforms to ensure the meaningful interpretation of data generated using those powerful tools.

## 1. Introduction

Myeloid malignancies in general are defined as clonal haemopoietic malfunction characterized by excessive proliferation, atypical self-renewal, and/or differentiation defects of haemopoietic stem cells (HSCs) and myeloid progenitor cells [1]. Leukaemia as a cancer category accounted for 440,000 new cases and more than 300,000 deaths worldwide in 2018 [2,3]. Acute myeloid leukaemia (AML) as one of the most devastating diseases in this category represents 1.2% of all new cancer cases in the U.S. in 2019 [4]. Our current understanding is that the onset of the disease is caused by different genetic and epigenetic changes in HSCs as well as functional changes in the bone marrow niche in the close vicinity of the emerging leukaemic cells. As a consequence of these developments a phenotypically distinct disease evolves that requires a specific treatment regimen depending on a multitude of risk factors [5]. Although some progress has been made specifically in the treatment of paediatric AML, the prognosis of adult AML specifically in the elderly patient has not improved significantly [6]. The high medical need is reflected in the increasing number of compounds in different stages of the development process: 1016 different drugs are currently investigated for their ability to tackle AML [7]. One important tool in this process are animal models as they are crucial for the selection of the most promising candidates at a late preclinical stage [8,9]. Furthermore, animal models are imminent for the understanding of the biology of the disease and thereby support the identification of potential new drug targets. Since the advent of the triple immunodeficient NOD-SCID-IL2rγ^null^ (NSG) and related mouse strains like NOD/Shi-scid-IL-2Rγ^null^ (NOG) the development of patient-derived leukaemia xenograft models was significantly facilitated [10,11]. Our group established a panel of 20 well-characterized acute leukaemia patient-derived xenograft models covering a broad range of different subtypes representing the molecular landscape of the disease. In the current study, we investigated the influence of the tumour microenvironment on different aspects of the leukaemic cell biology: the PDX were implanted via three different application routes and take rate, growth behaviour, expression of leukaemia-specific surface markers and sensitivity towards standard of care cytarabine was investigated. The implantation route had an impact explicitly on the tumour growth kinetics of an individual model. This was specifically interesting, as we identified this feature as a model intrinsic across different passages within one implantation scheme. Other model characteristics like the expression of three distinct surface markers and the take rate were model specific and stable across different engraftment sites. The sensitivity towards cytarabine was investigated in four models in a disseminated and the subcutaneous setting. In general, the sensitivity was not influenced by the implantation site. Nevertheless, using overall survival as a read-out, the sensitivity was influenced by the location of the tumour cells. In summary, the characteristics of an individual leukaemia PDX model are composed of the genetic make-up of the model itself and the tumour microenvironment modulated via the implantation route.

## 2. Results

### 2.1. Leukaemia PDX Retain the Cytogenetic Features of the Donor Patient and Mimic the Genomic Landscape of the Disease

Our group established and characterized 20 leukaemia PDX lines with the aim to develop a preclinical cancer model platform that recapitulates the human disease as close as possible to clinical reality. The collection included 18 acute myeloid leukaemia (AML), one acute promyelocytic leukaemia (APL) and one acute lymphoblastic leukaemia (ALL). The median age of the donor patients was 63 years (range 43–80 years), where 13 received no pre-treatment, 3 underwent peripheral blood stem cell transplantation (PBSCT) and 4 received other treatments mostly with a cytotoxic regimen. In the course of the establishment phase, we collected standard of care data for 19 out of 20 models. A molecular characterization was done on 16 models in total: eight models were analysed by whole exome sequencing (WES) and RNAsequencing (RNAseq), six models exclusively by WES and two models solely by RNAseq (see Table 1 and Tables S1 and S2). 

**Figure 1:**
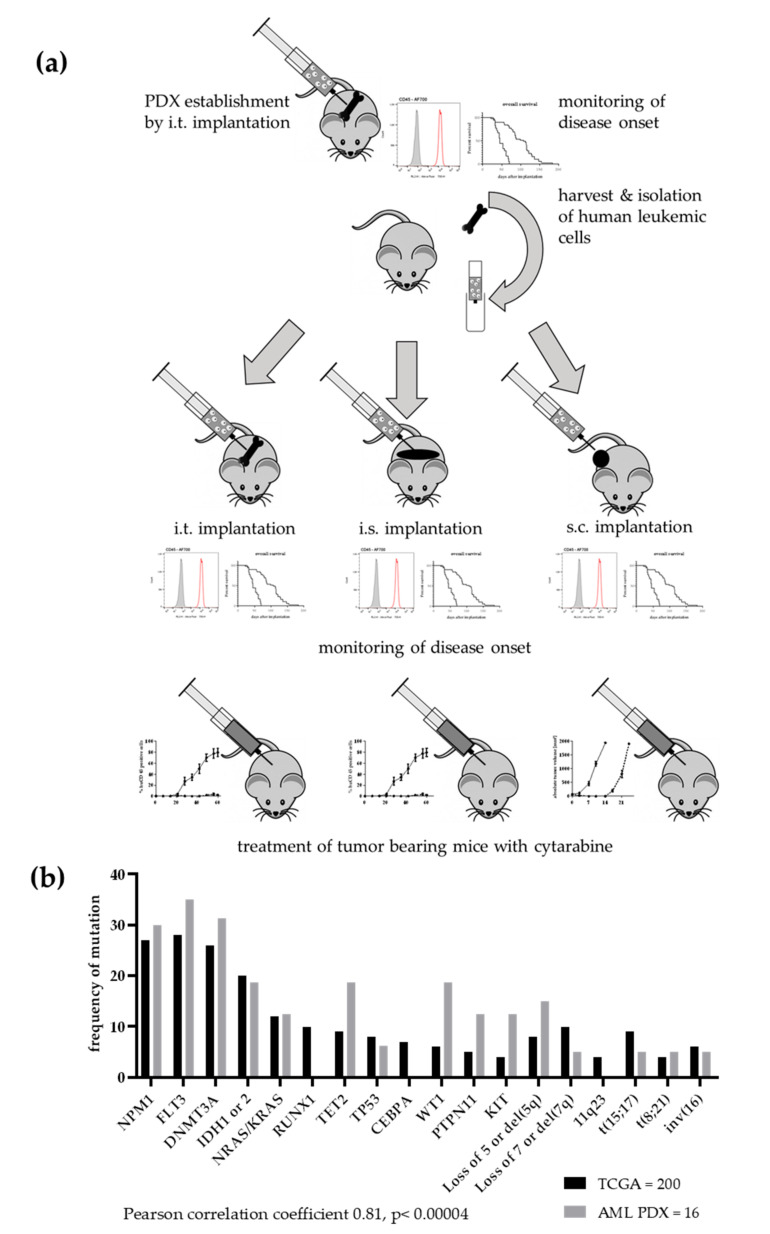
Establishment of a patient-derived xenograft panel of 20 acute leukaemia models. (**a**) Implantation scheme over several passages. Patient blasts were implanted into the bone marrow of NSG mice. When disease onset was observed by detection of human cells in peripheral blood and deterioration of overall condition, mice were sacrificed and cells from bone marrow and spleen harvested. After a mouse cell depletion human leukaemic cells were re-implanted into the bone marrow of recipient mice. This procedure was repeated until stable growth was observed. Subsequently, the PDX leukaemic cells were implanted into the bone marrow, in the spleen or subcutaneously into NSG mice. The monitoring of disease onset was performed as described before. Additionally, the antitumoral activity of cytarabine was evaluated in all three settings in four different PDX models. (**b**) For 16 models, mutational data were available. The comparison with TCGA data revealed a highly significant correlation between the two data sets. Mutational data were either acquired based on WES or RNAseq.

During the model establishment phase the patient-derived blasts were injected in the bone marrow of recipient mice via intratibial (i.t.) injection. Mice were monitored at this time of first passage for up to 18 months daily for clinical signs of leukaemia and every two weeks for detection of human leukaemic cells in the peripheral blood by flow cytometry (FC) analysis. When disease onset was perceived, mice were sacrificed and cells from the bone marrow and spleen harvested. After the depletion of mouse cells from the suspension, human leukaemic cells were re-implanted into the bone marrow of recipient mice. This procedure was repeated until stable growth was observed. A model was defined as established when stable growth over at least four passages, regrowth from liquid nitrogen and accordance between genetic data from the PDX with the data from the patient were obtained. To investigate the influence of the tumour microenvironment growth behaviour, take rate and expression of distinct surface markers were determined in twenty different models applied by three different implantation routes. Beside the i.t. approach, which was used during the establishment phase, the intrasplenic (i.s.) route and the subcutaneous (s.c.) implantation were validated in more detail. The detailed implantation scheme is described in Figure 1a.

The molecular analyses of the individual models were not only used to verify the origin of the PDX from a specific patient but also to determine how representative the leukaemia PDX panel was for a respective patient population. The comparison of the frequency of AML specific mutations in the PDX panel and data of an AML patient population from TCGA revealed a highly significant correlation of both data sets (Pearson correlation coefficient 0.81, *p* < 0.00004). Thus, our PDX panel largely represents the molecular landscape of the human disease (Figure 1b). 

### 2.2. The Overall Survival Time is a Model-Specific Feature which Evolves during the Establishment Phase of the Respective PDX. 

**Figure 2:**
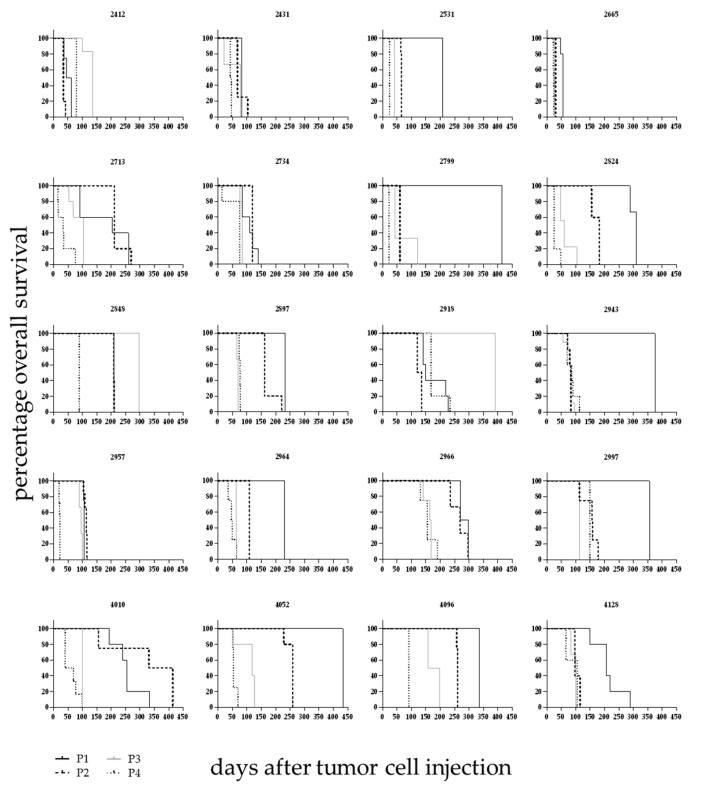
Overall survival in 20 leukaemia PDX models over different passages. Leukaemic blasts from donor patients were injected intratibially and mice monitored as described in detail in Figure 1 over the course of different passages. Until passage four the overall survival time changed between passages and stabilized at a model specific value afterwards.

Over the course of the development phase the individual PDX displayed differing overall survival times between the passages (Figure 2). Animals were sacrificed following stringent termination criteria enabling the comparison of those data across different models and passages. Within one model the first four passages differed significantly in terms of overall survival (OS, (Log-rank (Mantel–Cox) test, *p* < 0.005). From passage four on the OS time stabilized at a model specific value. The median OS time was characteristic for a specific established model and differed significantly between models. It ranged from 22 days (LEXF 2799) to 168 days (LEXF 2918) with a median OS time for the complete panel of 64.75 days (Table S3). 

### 2.3. The Implantation Site Has an Impact on the Overall Survival Time in a Number of PDX Models but not the Complete Panel

**Figure 3:**
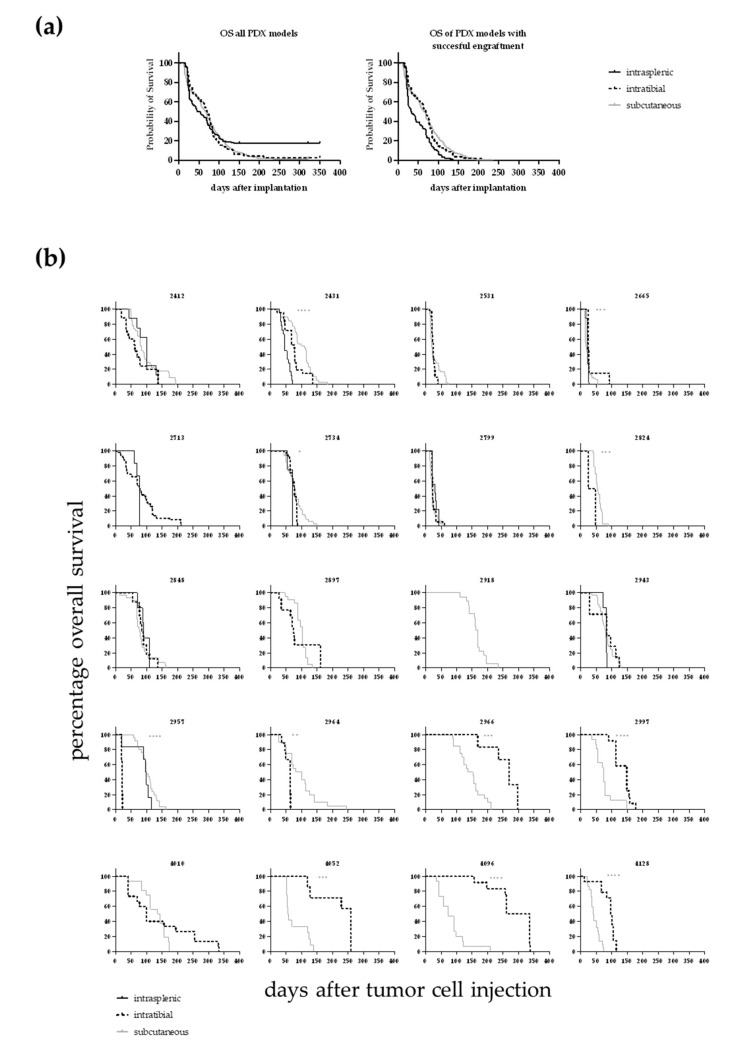
(**a**) Overall survival of 12 different leukaemia PDX dependent on the implantation route. In the case of the intrasplenic injection the outcome was influenced by the fact that three out of twelve tested models did not show any tumour growth during the observation period. In the left panel the OS is plotted for all models. The differences were statistically significant for i.s. vs. s.c.. In case only the models were taken into account which did show successful engraftment of leukaemic cells the i.s. implanted cells grew significantly faster than the s.c growing cells (right panel, all Log-rank (Mantel–Cox) test). (**b**) Overall survival determined in 20 leukaemia PDX models for up to three different injection sites. For 20 PDX models the overall survival rate was determined over time depending on the application route of the cancer cells. For 14 out of the 20 models a statistically significant difference between the implantation techniques was determined (Log-rank (Mantel–Cox) test).

The influence of the implantation site on OS was evaluated in 20 leukaemia PDX models. For all models the i.t. injection was compared with the s.c. approach, and for twelve models the i.s. implantation technique was additionally compared to the two other methods. In 14 out of 20 models the OS time was significantly influenced by the implantation site (Figure 3 and Table 2), whereas in six models no differences could be detected. We compared the median OS time across all models for which overall survival data in all tested settings was available (*n* = 12 models). In that case, the median OS of the i.s. implanted animals was 45 days (with three models showing no growth until end of the observation period). The median OS was 70 days for the i.t. injected mice and 67 days for the s.c. implanted animals. The differences were statistically significant for i.s. vs. s.c. and i.s. vs. i.t. In the case of the i.s. injection the outcome was influenced by the fact that three out of twelve tested models did not show any tumour growth during the observation period. Taking only models into account which showed successful engraftment of leukaemic cells the i.s. implanted cells grew significantly faster than the s.c growing cells (all Log-rank (Mantel–Cox) test).

### 2.4. The Implantation Site Has No Significant Impact on Take Rate but Does Influence the Infiltration Capacity in Haemopoietic Organs 

**Figure 4:**
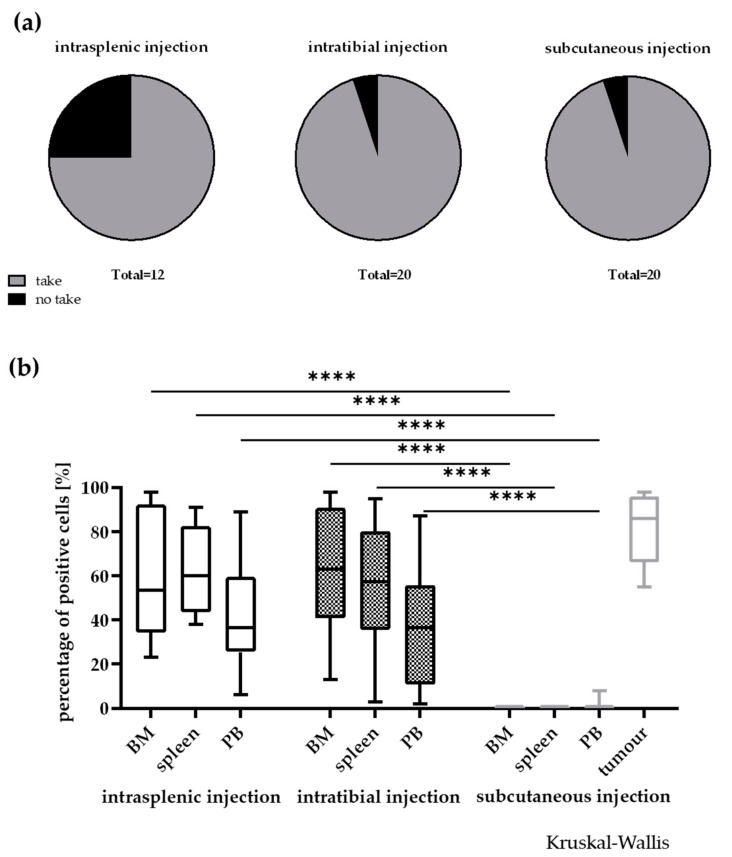
Influence of the injection site on take rate (**a**) and infiltration rate in different compartments (**b**) in 20 leukaemia PDX models. In general, the take rate was not influenced by the implantation site. Nevertheless, a few models did only engraft in two out of the three injection sites. A no take was defined by no growth after three individual experiments with *n* > 3 mice and an observation time >2-fold of the passaging time of the individual model. **** *p* < 0.0001.

In the course of the study we compared take rate and infiltration rate depending on the implantation techniques i.t., i.s. and s.c. In general, the take rate was very high in all models with individual models engrafting only in two out of three injection routes (Figure 4a). After i.s. injection three out of 12 models did not engraft. Nevertheless, if the engraftment was successful, the models grew significantly faster as in the i.t. or s.c. setting. One model, LEXF 2713, only engrafted reliably in a disseminated setting. This was vice versa for LEXF 2918 a reliable growth was specifically possible using the s.c. approach. Of note, this was the model with the longest OS time during the establishment phase. Overall, the differences in take rates were not statistically significant (two-way ANOVA). When mice reached termination criteria, BM, PB and spleen were harvested and analysed for infiltration of human leukaemic blasts by FC. Figure 4b plots the percentage of human CD45+ cells over all models in the three different compartments over three implantation techniques. With the exception of one model, LEXF 2431, the s.c. implanted leukaemia cells did not circulate in PB or infiltrated the investigated haemopoietic organs. As a consequence, the infiltration rates of the disseminated approaches in BM, PB and spleen were significantly higher compared to the s.c. method (*p* < 0.0001, Kruskal–Wallis test). For the two disseminated approaches the infiltration pattern was similar in BM, PB and spleen. Comparing the infiltration rate at the respective injection sites between the three settings also revealed no statistical differences (Kruskal–Wallis test).

### 2.5. Individual Leukaemia PDX Express a Specific Surface Marker Pattern which is Stable across Different Engraftment Sites

**Figure 5:**
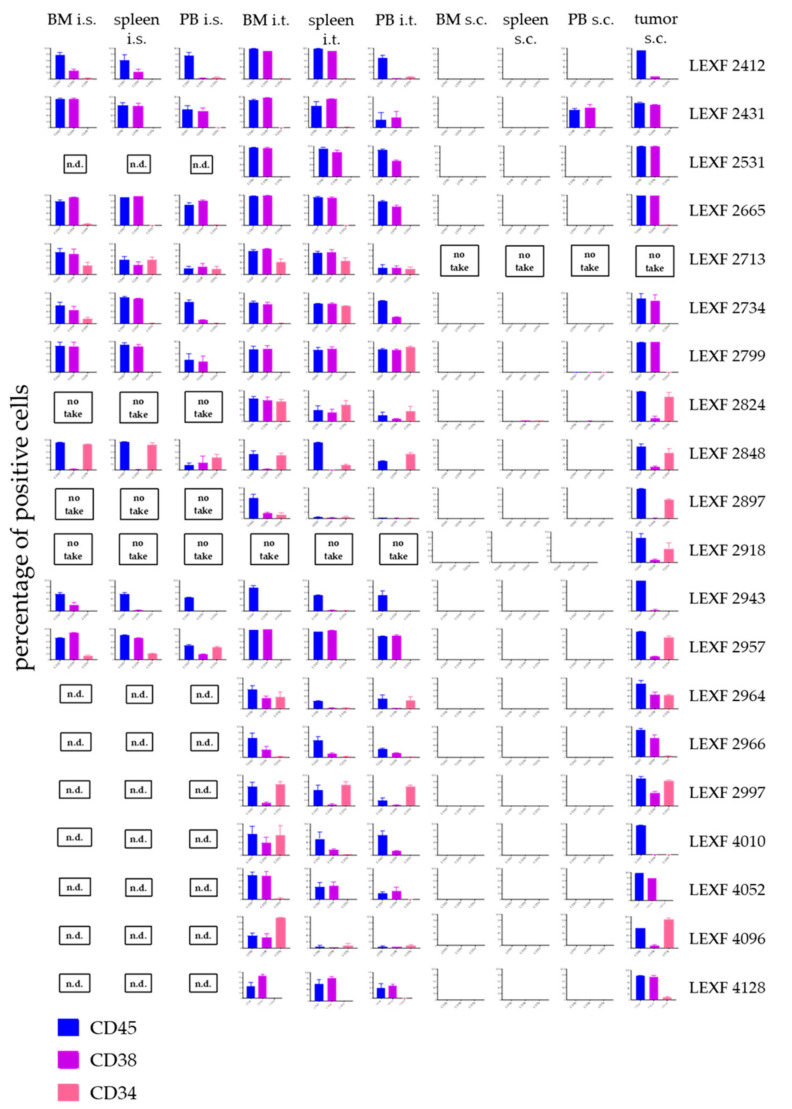
Infiltration pattern of 20 leukaemia PDX in bone marrow, spleen and peripheral blood depending on the injection site of the leukaemic cells. Each PDX model displayed its specific expression pattern of the three investigated surface markers CD45, CD38 and CD34 in all tested settings. Despite for one model, LEXF 2431, leukaemic cells did not infiltrate the haemopoietic organs when injected subcutaneously. A no take was defined by no growth after three individual experiments with n > 3 mice and an observation time > 2-fold of the passaging time of the individual model. n.d., not determined.

During the development phase of the PDX models, it was already observed that individual models expressed specific surface marker patterns, which were stable across the different passages. In the current study we investigated whether this was also the case when the PDX were implanted via different routes. Figure 5 gives an overview of the percentage of positive cells determined by FC for human CD45, CD34 and CD38 as the most ubiquitously expressed surface markers in our leukaemia PDX panel. The expression pattern was stable and specific across the different compartments and injection routes. A cluster analysis was performed and results were plotted as a heatmap (data not shown) and as a principle component analysis plot (Figure S1). Neither the injection route (i.t vs. i.s. vs. s.c) nor the engraftment site (BM vs. spleen vs. PB) displayed a strong correlation coefficient. Data from individual models showed correlation coefficients > 0.5 (*p* < 0.05) whereas between different models the data sets did not correlate (Pearson correlation)). 

### 2.6. Cytarabine is Active in Four Leukaemia PDX Models with Varying Degree of Efficacy Depending on the Model and the Implantation Site

**Figure 6:**
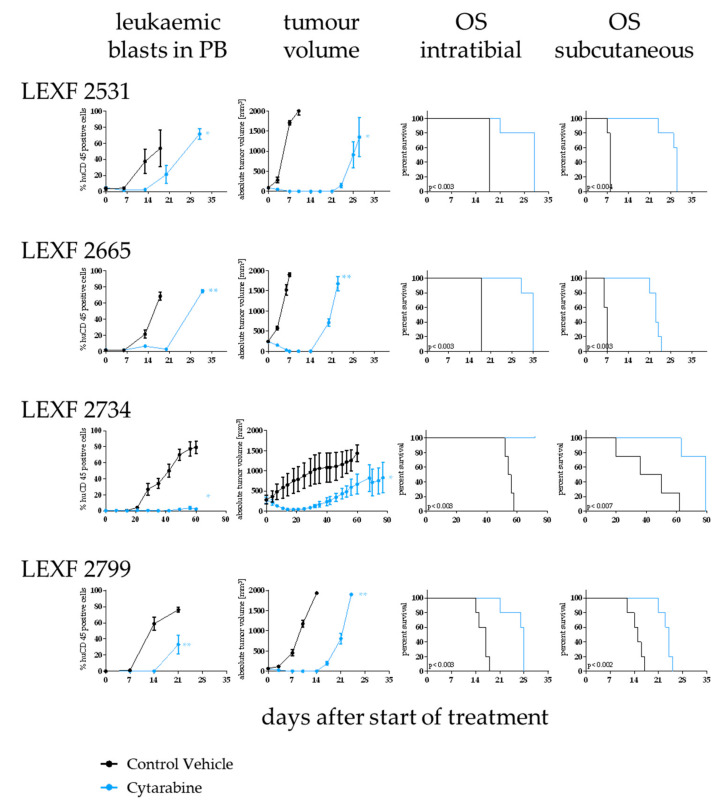
Determination of sensitivity towards cytarabine in four leukaemia PDX models depending on the implantation route of the tumour cells. Antitumor activity was determined by percentage of leukaemic blasts in peripheral blood (i.t. injected animals), tumour volume (s.c. implanted animals) and overall survival (i.t. and s.c.). Cytarabine was active an all tested models and settings although with varying degree depending on the model and the implantation site. Significance was calculated using the Log-rank (Mantel–Cox) test for OS and Mann–Whitney for tumour volume and percentage of human CD45+ cells.

Cytarabine as a first line treatment in acute leukaemia was tested in four PDX models implanted i.t. and s.c., respectively. The antitumoral activity was determined by OS and percentage of human CD45+ cells in PB (i.t.) and absolute tumour volume in mm ³ (s.c.). Overall, cytarabine showed statistically significant activity in all four models independent of the injection route. The optimal test/control (T/C) values and their statistical significance of an individual model were similar between the two experimental set-ups (Figure 6). Nevertheless, when comparing the influence of the treatment on OS, only LEXF 2799 showed analogous growth kinetics. Cytarabine induced a 1.6fold increase in OS in the i.t. as well as the s.c. experiment. In LEXF 2665 (3.29fold vs 1.94fold) and LEXF 2531 (3.75fold vs 1.72fold) the prolongation of OS was more pronounced in the s.c. setting. In contrast, animal bearing LEXF 2734 in a disseminated mode benefit more from the treatment with cytarabine as mice bearing the same tumour model s.c. (1.84 fold vs complete remission). In the respective donor patients of the leukaemia PDX models cytarabine achieved a complete remission in the course of the first line treatment. Three of four patients underwent a relapse and received additional therapies during the course of their disease. Thus, despite the minor differences in the duration of the antitumoral effect, the models reflected the clinical response of the donor patient regardless of the implantation site.

## 3. Discussion

Rodent tumour models have been generated since the 1960s, with xenografts of human tumour models emerging in the 1980s [12]. The development of the nu/nu mutant mouse lacking functional thymus tissue and therefore T cells enabled the engraftment and serial transplantation of human tumour tissue into mice [13]. With the development of more immunosuppressive mouse strains like SCID, NOD/SCID or most recently NOD/SCID/IL2Rγnull (NOG and NSG) mice the engraftment rate of human tumour tissue improved significantly over the last 30 years. Notably the latter enabled not only an improved engraftment of solid tumours but the establishment of PDX from haematological malignancies namely acute leukaemia and different types of lymphomas [14,15]. 

Recently, there have been multiple efforts in industry as well as academia to establish large panels of well characterized PDX models covering a wide range of different tumour types [16]. Currently, these collections represent a predictive preclinical tool for drug development as well as tumour biology research displaying most consistently the complexity of tumour heterogeneity and molecular diversity of human cancers. 

We established a panel of 20 acute leukaemia PDX models recapitulating the diversity of the disease. The models were established by injection into the bone marrow of recipient mice mimicking the bone marrow niche in the patient as close as possible. The i.t. injection was previously described as a successful implantation technique for other haematological malignancies using established cell lines and patient-derived material [17,18,19]. To the best of our knowledge the current study describes for the first time this method in a systematic approach to establish a panel of patient-derived acute leukaemia models. 

The comparison with TCGA data revealed a high correlation with the patient cohort indicating that the PDX panel mirrored clinical reality to a great extent. The comparison with published data in this context led to the assumption that besides the engraftment technique and the extended observation period in the establishment phase [20], the choice of the mouse strain is crucial [21,22,23]. Over the course of the establishment phase the overall survival time developed into a model specific feature, which was used in the following experiments as an additional read-out besides the infiltration rate in haemopoietic organs. 

To understand the influence of the microenvironment more in detail and to further evaluate different implantation techniques, we compared three implantation routes: the already established i.t. vs. i.s. and s.c. Take rate, infiltration rate, OS and sensitivity towards standard of care cytarabine were compared depending on the injection route. OS was significantly influenced by the engraftment site in 14 out of 20 models. The models in the two groups (OS dependent on injection site vs. non-dependent) displayed no obvious discrimination criteria. Phenotypical and molecular characteristics as well as clinical features from the donor patient were evenly distributed across both groups. Thus, the mechanistic background for this phenomenon remains to be elucidated in future studies. Of note, it has to be taken into account, that due to the nature of the different growth patterns the termination criteria had to be adapted to the tumour growth and might have an impact on differences in OS across different implantation techniques as well. Other groups reported a correlation between patient related characteristics and engraftment latency in the stage of establishment [24]. Meyer et al. [25] systematically investigated the different factors affecting human leukaemia engraftment in mice: the transplantation procedure was one relevant factor they identified as key for the engraftment kinetics. Interestingly, the implantation site had no significant impact on the take rate, although after i.s. implantation three models displayed no engraftment over the course of the experiment. Wang et al. [26] reported the successful engraftment of five out of eleven cases of human all after i.s. injection. This was in line with our observation of nine out of twelve successful engraftments. However, the major difference between the two studies is that Wang et al. [26] compared the engraftment capacity of patient material while our group used PDX models established by i.t. injection.

In our hands, the human leukaemic cells specifically infiltrated the haemopoietic organs when injected i.t. or i.s. Only one s.c. model exhibited a reproducible engraftment of human leukaemic cells in the PB. Of note, no leukaemic cells could be detected in the bone marrow and spleen of those animals. Yan et al. [27] reported the infiltration of haemopoietic organs in animals bearing s.c. implanted leukaemia PDX depending on the growth behaviour of the respective model. The differences in the outcome of those two studies might be mainly related to the fact that different mouse strains were used. The SCID mice used by Yan et al. [27] allowed, due to their higher immunological competence, engraftment of only very aggressive forms of leukaemia. In contrast the NSG used in the current study enabled the engraftment of less aggressive disease forms that do not tend to disseminate in the unfavourable environment of a s.c. implantation. 

The leukaemia PDX models were characterized by a definite expression pattern of surface markers determined by FC: CD45, CD34 and CD38 were expressed in a model specific percentage on the tumour cells. The implantation site did not alter the expression of those markers significantly as shown by the correlation and principle component analysis. To the best of our knowledge, similar analyses were not published until now. In a slightly different context, Vick et al. [14] reported that the immunophenotype was stable across different passages, which is completely in line with our observations that the immunophenotype is a model inherent feature. 

In four of the leukaemia models we evaluated the antitumoral activity of cytarabine in relation to the application route of the tumour cells. Cytarabine achieved a complete eradication of the tumour cells from peripheral blood and the subcutaneous injection site (if applicable) in all experiments independent of the model or the growth pattern. This reflected very well the patient’s outcome, as all four donor patients underwent complete remission under cytarabine treatment. Tumour growth kinetics after the treatment cycle has ended was impacted by the implantation site in three out of four models. Two models displayed an elongated remission phase in the s.c. setting whereas in one model the opposite was observed. Despite the fact that the database with only one compound in four models was rather small, the results do underline the predictive power of leukaemia PDX models. Our results were in line with studies from other groups investigating the predictivity of PDX for future treatment options in the clinic [28,29,30]. 

Taken together, all three investigated implantation techniques delivered reliable results and are feasible for preclinical studies in the drug development as well as tumour biology context. Nevertheless, the distinct differences between individual data sets highlight the need for well characterized preclinical models to ensure the meaningful interpretation of data generated using those powerful tools.

## 4. Materials and Methods 

### 4.1. PDX Establishment

T-and B-cell depleted peripheral blood or bone marrow cells (3 × 10 ^6^ cells/mouse) from leukaemia patients were injected intratibially (i.t.) in the bone marrow of 4–6-week old NSG (NOD/Shi-scid/IL-2Rγnull; Charles River, France) mice. No pre-conditioning with irradiation or chemotherapeutic based treatment was performed prior to injection. Human T-and B-cells were depleted by MACS selection using CD3 and CD19 microbeads (#130-050-101 and #130-050-301, Miltenyi Biotec, Germany) according to the manufacturer’s instructions. Leukaemic cell engraftment was determined by flow cytometry (FC) in bone marrow (BM), peripheral blood (PB) and spleen during the course of the experiment and at the end of the study. Overall survival (OS) served as an additional read-out. The PDX model was defined as established when stable growth over at least four passages and regrowth from liquid nitrogen was observed. All surgery was performed under isoflurane anaesthesia, and all efforts were made to minimize suffering.

### 4.2. Comparison of Different Injection Routes

Mouse-cell depleted human leukaemic blasts (3 × 10 ^6^ cells/mouse) from established leukaemia PDX were injected into the spleen (i.s.), i.t. or subcutaneous (s.c.) in 4–6-week old NSG (Charles River, France) mice. No pre-conditioning with irradiation or chemotherapeutic based treatment was performed prior to injection. Mouse cell depletion was performed using the mouse cell depletion kit (#130-104-694, Miltenyi Biotec, Bergisch Gladbach, Germany) [31]. The i.s. injection was done via surgical intervention. Under isoflurane anaesthesia and analgesic treatment a small (3 mm) incision was made in the skin and the peritoneum. The leukaemia cells (3 × 106 cells) were injected in a total volume of approx. 30 µL. The puncture canal was closed and haemostasis was carried out using sterile cotton swabs. Skin and peritoneum were closed using resorbable suture material and sore clips. Leukaemic cell engraftment was determined by FC in BM, PB, spleen and subcutaneously growing tumour mass at the end of a study. OS served as an additional read-out. For subcutaneously implanted mice tumour volume was determined twice weekly using electronic calipers and engrafted mice were sacrificed when a tumour volume of 1.800 mm³ was gained. For the disseminated growing tumours, the animals were sacrificed when disease onset was observed by detection of human cells in peripheral blood by FC (> 2%) and deterioration of overall condition indicated by a body weight loss of > 15% over the course of 2 days. All surgery was performed under isoflurane anaesthesia, and all efforts were made to minimize suffering.

### 4.3. Flow Cytometry Analyses

BM, spleen, tumour or PB cells (5 × 10^5^ to 1 × 10^6^) from tumour bearing animals were harvested and incubated with the primary antibody: hCD45 anti human PB (MHCD4528; Invitrogen, Carlsbad, CA, USA), mCD45.1 anti-mouse PE (553776; BD Biosciences, Franklin Lakes, NJ, USA), hCD34 anti-human FITC (555821; BD Biosciences), hCD38 anti-human BV605 (562665; BD Biosciences), hCD33 anti-human APC (551378; BD Biosciences), hHLA-ABC-PECy5 (555554; BD Biosciences), hCD3-FITC anti-human FITC (555916; BD Biosciences) or isotype control and the mean fluorescence intensity was analysed by flow cytometry. The tumour load was assessed by measurement of the percentages of cells positive for different human surface markers after red blood cell lysis with ammonium chloride. Staining was performed in the presence of CD16/CD32 Abs to block non-specific staining (rat anti-mouse CD16/CD32 (FcγIII/II) receptor IgG2b, BD Pharmingen, San Diego, CA, USA). Samples were analysed on an Attune Acoustic Focusing Cytometer NXT (Applied Biosystems, Waltham, MA, USA) which recorded 50,000 events. 

### 4.4. Standard of Care Testing

Mice were treated with cytarabine (15 mg/kg/day given intravenously; STADApharm, Bad Vilbel, Germany) or vehicle (PBS) for five consecutive days. Treatment commenced upon disease onset: for i.t. implanted animals this was determined as > 2% human CD45+ cells in PB. For s.c. implanted animals the starting median absolute tumour volume was 100 mm³. Beside those read-outs OS served as a read-out for both implantation techniques. The relative volume of an individual tumour on day X (RTVx) was calculated by dividing the absolute volume (mm^3^) of the respective tumour on day X (Tx) by the absolute volume of the same tumour on the day of randomization, i.e., on day 0 (T0), multiplied by 100, as shown by the following equation: RTVx [%] = Tx/T10 × 100. Tumour inhibition on a particular day (T/Cx) was calculated from the median RTV of a test group and the median RTV of a control group multiplied by 100, as shown by the following equation: T/Cx [%] = median RTVx treated group/median RTVx control group*100. The minimum T/Cx [%] value recorded for a particular group during an experiment represented the maximum anti-tumour activity for the respective compound and were thereby defined as optimal T/C. All experiments were performed according to the relevant animal welfare guidelines published by FELASA and GV-SOLAS in an AAALAC accredited animal facility. Tumour volume and body weight were determined twice per week. Percentage of human CD45+ cells in PB once a week. The calculation of the optimal T/C value was performed as described above for tumour volume. 

### 4.5. Ethics Statement

This study was carried out in strict accordance with the recommendations in the Guide for the Care and Use of Laboratory Animals of the Society of Laboratory Animals (GV SOLAS) in an AAALAC accredited animal facility. All animal experiments were approved by the Committee on the Ethics of Animal Experiments of the regional council (Regierungspräsidium Freiburg, Abt. Landwirtschaft, Ländlicher Raum, Veterinär- und Lebensmittelwesen - Ref. 35, permit-#: G-09/59, G-12/86, G-17/138). The analyses were performed according to the guidelines of the Declaration of Helsinki and good clinical practice. All patients gave their written informed consent for institutional-initiated research studies and analyses of clinical outcome studies conforming to our institutional review board guidelines. All experiments were approved by the ethical commission of the Albert Ludwig University Freiburg (permit-# EK Freiburg: 279/10, 07.09.2010). 

### 4.6. Statistical Analysis

Student’s *t* test, two-tailed and one-way ANOVA followed by Tukey’s multiple comparisons test were used to calculate all reported p-values. Log-rank [Mantel–Cox]-test was used to calculate overall survival. Descriptive analyses were assessed whenever appropriate and were obtained using GraphPrism software [32] 

### 4.7. Molecular Analysis and Comparison with TCGA Data

The mutational data of the PDX models were retrieved via the online compendium from Charles River [33] and compared to the mutational data from TCGA via the TCGA data portal [34]. The frequency of mutation was compared between both data sets.

### 4.8. Cluster Analysis

Heat maps and PCA plots were generated using the ClustVis web tool [35]. Centering and unit variance scaling was applied to the sample ID. Both surface markers and sample IDs were clustered using correlation distance and average linkage. Hierarchical clustering of the heatmap started with calculating all pairwise distances. Objects with the smallest distance were merged in each step. The clustering method defined how to go from object level to cluster level when calculating distance between two clusters. The clustering distance was defined as Pearson correlation subtracted from 1.

## 5. Conclusions

We took advantage of our well characterized leukaemia PDX panel and evaluated the influence of the injection site on different parameters of the PDX growth behaviour. Taken together, the features of an individual PDX model are dictated by the genetic make-up of the model itself and the tumour microenvironment modified in the current study via the engraftment site. Finally, the question of which in vivo model will most precisely reflect the patient situation ultimately may depend on the focus of the scientific question.

## Figures and Tables

**Table 1:** Characteristics of the leukaemia patient-derived xenografts (PDX) panel.

Nr of Models	pts Data								PDX Data		
	Age, Median (Range)	Gender (f:m)	Pre-Treatment		Subtype		Mutational Status		SoC Data Available	WES Data Available	RNAseq Data Available
20	63 (43–80) years	11:9	none	13	AML	18	FLT3-ITD	6	19	14	10
			PBSCT	3	ALL	1	FLT3-TKD	1			
			other	4	APL	1	NPM1-A	6			

Abbreviations: SoC, standard of care; WES, whole exome sequencing; RNAseq, RNA sequencing.

**Table 2:** Comparison of overall survival (OS) times within one model depending on the implantation route (Log-rank (Mantel–Cox) test).

Model Name	Intrasplenic vs. Intratibial	Intrasplenic vs. Subcutaneous	Intratibial vs. Subcutaneous
LEXF 2412	n.s.	n.s.	n.s.
LEXF 2431	<0.0001	<0.0001	0.0091
LEXF 2531	n.d.	n.d.	n.s.
LEXF 2665	<0.0001	n.s.	0.0002
LEXF 2713	<0.0001	<0.0001	<0.0001
LEXF 2734	0.0334	n.s.	0.0208
LEXF 2799	n.s.	n.s.	n.s.
LEXF 2824	<0.0001	<0.0001	0.0001
LEXF 2848	n.s.	n.s.	n.s.
LEXF 2897	0.0002	<0.0001	n.s.
LEXF 2918	n.s.	<0.0001	<0.0001
LEXF 2943	n.s.	n.s.	n.s.
LEXF 2957	0.0009	n.s.	<0.0001
LEXF 2964	n.d.	n.d.	0.001
LEXF 2966	n.d.	n.d.	0.0003
LEXF 2997	n.d.	n.d.	<0.0001
LEXF 4010	n.d.	n.d.	n.s.
LEXF 4052	n.d.	n.d.	0.0006
LEXF 4096	n.d.	n.d.	<0.0001
LEXF 4128	n.d.	n.d.	<0.0001
all models	n.s.	0.049	n.s
all models w/o no takes	<0.0001	<0.0001	n.s.

Abbreviations: n.d: not determined; n.s: not significant.

## Supplementary Materials

The following are available online at https://www.mdpi.com/2072-6694/12/5/1349/s1, Figure S1: Principle component analysis of the expression pattern of three different surface markers in 20 leukaemia PDX models depending on the application route and engraftment site, Table S1: patient characteristics of 20 leukaemia PDX models, Table S2: Mutational landscape of acute leukemia PDX models. Table S3: Statistical analysis of overall survival time of 20 established leukaemia PDX implanted intratibially in NSG mice.
