# Supplementary material for: Impact of the Injection Site on Growth Characteristics, Phenotype and Sensitivity towards Cytarabine of Twenty Acute Leukaemia Patient-Derived Xenograft Models
Source: Cancers (Basel). 2020 May 25;12(5):1349. doi: 10.3390/cancers12051349 (PMC7281503; doi:10.3390/cancers12051349)
Supplement: Supplementary file 1 [file cancers-12-01349-s001.pdf]

Supplemental Materials: Impact of the Injection Site on Growth Characteristics, Phenotype and Sensitivity towards Cytarabine of Twenty Acute Leukaemia Patient-Derived Xenograft Models

Julia Schueler, Gabriele Greve, Dorothée Lenhard, Milena Pantic, Anna Edinger, Eva Oswaldand and Michael Lübbert

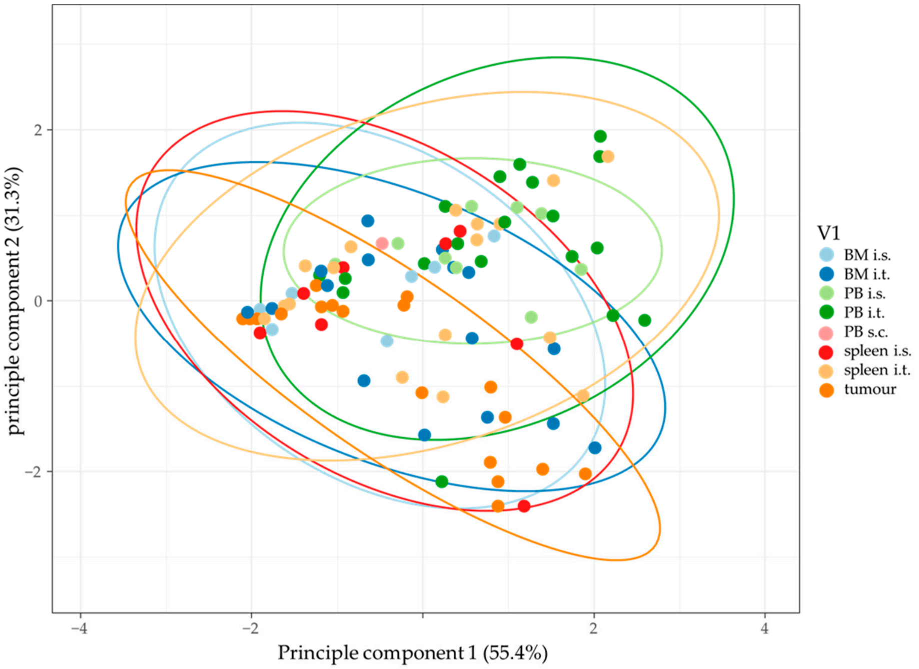

Figure S1. Principle component analysis of the expression pattern of three different surface markers in 20 leukaemia PDX models depending on the application route and engraftment site. Non-engrafted compartments were not included in the computation. Neither the application route, nor the engraftment site had a major impact on the expression pattern of the three investigated markers.

Table S1: patient characteristics of 20 leukaemia PDX models.

	WBC [thous/ µl]	BM blasts [%]	Diagnosis	age [y]	gender	mutational status			pre-treatment
						NPM1	FLT3	karyotype	
LEXF 2412	43.7	90	AML	55	f	NPM1-A	FLT3-ITD	46,XX	none
LEXF 2431	1.5	62	AML	74	f	wt	wt	46,XX	none
LEXF 2531	3.0	80	AML	46	f	wt	wt	47; XX, +21 [19], 46, XX [1]	Daunorubicine, Cytarabine, PBSCT, Rituximab, Busulfan, Cyclophosphamide
LEXF 2665	13.6	30	ALL	70	f	n.d.	n.d.	47,XX,+8[7]; 46,XX[14]	Dexamethasone
LEXF 2713	3.7	22	AML	43	f	wt	wt	47,XX,del(2)(p13),der(9)t(2;9)(p13;q34),+der(9)t(2;9)(p13;q34),t(9;16;20;17)(q21;q21;?p13;q21)[7] 47,XX,del(2)(p13),der(9)t(2;9)(p13;q34),+der(9)t(2;9)p(13;q34),t(9;16;20;17)(q21;q21;?p13;q21),ider(13)(q10)del(13)(q13q31)[6] 46,XX,del(2)(p13),der(5)t(5;18)(q31;?q21),+8,der(8)t(8;12)(q11;q24),der(8)t(8;20)(q22;q13),der(9)t(2;9)(p13;q34),.der(9)t(2;9)(p13;q34),der(9)t(9;17)(q21;q21),der(12)t(8;12)(q24;q24),der(16)t(9;16)(q21;q21),-17,-18,der(20)t(5;20)(q31;q13),der(20)t(16;20)(q21;?p13) [6] 46,XX [1]	none
LEXF 2734	139.7	96	AML	59	m	mut	FLT3-ITD	46, XY, del(11)(p13p16) [19], 46,XY [3]	Daunorubicine, Cytarabine, PBSCT, Fludarabine, BCNU, Melphalan, Sorafenib, DLI
LEXF 2799	3.6	25	AML	53	m	wt	wt	47,XY,**+8**,del(9)(q22q34)[9]/ 46,XY[2];	none

LEXF 2824	2.4	22	AML	80	m	n.d.	n.d.	39, XY, del(3)(p14p26), -4, der(5)t(5;8)(q11;?), -6, der(8)(18qter->18q21::6q22q14 ->6q24::8p23->8p12::6?->6?::8p12->8qter), del(8)(q23q24), +der(9)(9pter->9p24::9p12->9q31::18q12->18q22::13q?->13q?::15q21->15qter), der(9;18)(9pter->9p10::18q10->18q22::13q?->13q?::21q22->21q22::hsr::15q?->15q?::21q22->21q22::15q24->15qter), der(13;13)(q10;q10), -15, r(16)(p12q12), -17, der(17)(17pter->17q12)::hsr::17q12->17q25::17q11->17qter), -18, der(19)(4pter->4p11::19?13->13?13::hsr::19?ter), -21 [6]<br>46,XY [5]	none
LEXF 2848	40.1	100	AML	45	m	wt	wt	46,XY,ish ins(16)(8q22p13p13) 16p13(MYH11+)16q22(CBFB+,MYH11+)[16]; 46,XY[4]	none
LEXF 2897	48.1	85	APL	57	f	wt	FLT3-ITD	46,X,-X,+8,del(9)(q21q34),t(15;17)(q24;q21) [20]	none
LEXF 2918	8.8	80	AML	62	m	n.d.	wt	45,XY,-7,+8, t(12;22)(p13;q12)	BCNU, Fludarabine, Melphalan, PBSCT, Cyclosporin A, Prednisolone, ECP
LEXF 2943	44.4	60	AML	80	f	wt	wt	46,XX	none
LEXF 2957	49.4	4	AML	73	m	wt	FLT3-ITD	46,XY,del(17)(p11p13),der(18)t(1 ;18)(q32;p11) [15]<br>46,XY,+1,der(1 ;15)(q10;q10),del(17)(p11p13) [2]<br>46,XY [3]	Decitabine, ATRA
LEXF 2964	1.1	15	AML	72	m	wt	wt	45,XY,+Y,del(5)(q12q 3),-18,der(20;22)(p10;q10),+der(20;22X21qter->21q11 ::20q13->20q1 :20q11->20q10::20q10->20q11::20q13->20q13::22p11->22qter),-21 [3]<br>46,XY (21]	Decitabine
LEXF 2966	2.1	30	AML	61	f	mut	wt	47,XX,+8[2]/46,XX[21]	none

LEXF 2997	2.1	11	AML	64	m	wt	wt	45,XY,t(4;15)(q23;q26),del(5)(q23q34),del(6)(q14q22-23),-7[10]	Azacitidine
LEXF 4010	11.8	62	AML	64	f	mut	FLT3-TDK	46,XX	none
LEXF 4052	1.5	30	AML	63	f	wt	wt	44,XX,-3,-4,del(7)(q11), der(10)(:p13> 1 Oq26::?: :17q21>17qter),del(12) (q15),der(13)t(3;13)(q13;q14),del(16)(q2 4),-17,-17,der(18)t(17;18)(q21;q21), +3mar[19]/46,XX[1]	none
LEXF 4096	48.8	75	AML	58	m	mut	FLT3-ITD	46,XY[4]	none
LEXF 4128	238.5	90	AML	75	f	mut	FLT3-ITD	46,XX [21]	none

Abbreviations: ATRA, All-trans retinoic acid; BCNU, 1,3-bis (2-chloroethyl)-1-nitroso-urea; DLI, donor lymphocyte infusion; ECP, extracorporeal photochemotherapy; f, female; m, male; mut, mutated; n.d., not determined; PBSCT, peripheral blood stem cell transplantation; WBC, white blood cell count; wt, wild type

Table S2. Mutational landscape of acute leukemia PDX models.

	CEBPA	DNMT3A	IDH1	IDH2	KIT	KRAS	NRAS	PTPN11	RUNX1	TET2	TP53	WT1	FLT3	Loss of 5 or del(5q)	Loss of 7 or del(7q)	11q23	t(15;17)	t(8;21)	inv(16)
LEXF 2412	wt	wt	wt	wt	wt	wt	wt	wt	wt	V1718L	wt	frameshift_var	FLT3-ITD	no	no	no	no	no	no
LEXF 2431	wt	wt	wt	wt	wt	wt	wt	S506P	wt	wt	wt	wt	wt	no	no	no	no	no	no
LEXF 2531	wt	wt	wt	wt	wt	wt	wt	wt	wt	wt	wt	wt	wt	no	no	no	no	no	no
LEXF 2665	wt	wt	wt	wt	wt	wt	wt	wt	wt	wt	wt	wt	FLT3-ITD	no	no	no	no	no	no

LEXF 2713	wt	wt	wt	wt	wt	wt	wt	wt	wt	wt	wt	wt	wt	no	no	no	no	no	no
LEXF 2734	wt	R882H	R132H	wt	wt	wt	wt	wt	wt	wt	wt	wt	FLT3-ITD	no	no	no	no	no	no
LEXF 2799	wt	wt	wt	wt	wt	wt	wt	wt	wt	wt	wt	wt	wt	no	no	no	no	yes	no
LEXF 2824	wt	P904L	wt	wt	wt	wt	wt	wt	wt	wt	wt	wt	wt	no	no	no	no	no	no
LEXF 2848	wt	wt	wt	wt	D816Y	wt	wt	wt	wt	wt	wt	wt	wt	no	no	no	no	no	yes
LEXF 2897	wt	wt	wt	wt	wt	wt	wt	wt	wt	wt	wt	wt	FLT3-ITD	no	no	no	yes	no	no
LEXF 2918	wt	wt	wt	R140Q	wt	wt	G13C	wt	wt	wt	wt	wt	wt	no	no	no	no	no	no
LEXF 2943	wt	wt	wt	R140Q	wt	wt	wt	wt	wt	wt	wt	wt	wt	no	no	no	no	no	no
LEXF 2957	wt	inframe_insertion	wt	wt	wt	wt	G12D	wt	wt	stop_gained	wt	stop_gained,frameshift_var	FLT3-ITD	del(5)(q12q 3)	no	no	no	no	no
LEXF 2964	no data available																		
LEXF 2966	wt	R882P	wt	wt	wt	wt	wt	wt	wt	R1261H|frameshift_variant	wt	R434H	wt	no	no	no	no	no	no
LEXF 2997	wt	wt	wt	wt	V399I|D816Y	wt	wt	wt	wt	wt	R282W	wt	wt	del(5)(q23q34)	no	no	no	no	no
LEXF 4010	no data available																		

LEXF 4052	no data available																		
LEXF 4096	no data available																		
LEXF 4128	wt	R882C	wt	wt	wt	wt	wt	M520V	wt	wt	wt	wt	FLT3-ITD	no	del(7)(q11)	no	no	no	no

Table S3. Statistical analysis of overall survival time of 20 established leukaemia PDX implanted intratibially in NSG mice.

	**LEXF 2412**	**LEXF 2431**	**LEXF 2531**	**LEXF 2665**	**LEXF 2713**	**LEXF 2734**	**LEXF 2799**	**LEXF 2824**	**LEXF 2848**	**LEXF 2897**	**LEXF 2918**	**LEXF 2943**	**LEXF 2957**	**LEXF 2964**	**LEXF 2966**	**LEXF 2997**	**LEXF 4010**	**LEXF 4052**	**LEXF 4096**	**LEXF 4128**	**median OS**
**LEXF 2412**		0.0189	0.0082	0.0148	0.0136	0.0141	0.0143	0.0082	0.0455	0.0246	0.0082	n.s.	0.0103	0.0177	0.0143	0.0143	n.s.	0.0169	0.0253	n.s.	**79**
**LEXF 2431**	0.0189		0.0047	0.0067	n.s.	n.s.	0.0082	n.s.	0.0446	0.0189	0.0039	0.0039	0.0043	n.s.	0.0084	0.0084	n.s.	0.0084	0.0189	0.0039	**44**
**LEXF 2531**	0.0082	0.0047		n.s.	n.s.	n.s.	0.0047	0.0027	0.0143	0.0082	0.0027	0.0027	0.0019	0.0047	0.0047	0.0047	0.0016	0.0047	0.0082	0.0027	**24**
**LEXF 2665**	0.0148	0.0067	n.s.		n.s.	n.s.	n.s.	0.0031	0.0343	0.0148	0.0031	0.0031	0.017	0.0067	0.0067	0.0067	0.0015	0.0067	0.0148	0.0031	**24**
**LEXF 2713**	0.0136	n.s.	n.s.	n.s.		n.s.	n.s.	n.s.	0.0405	n.s.	0.0018	0.0153	n.s.	n.s.	0.0049	0.0049	0.0407	n.s.	0.0136	0.0153	**33**
**LEXF 2734**	0.0141	n.s.	n.s.	n.s.	n.s.		n.s.	0.0404	0.0304	n.s.	0.0035	n.s.	0.0332	0.0483	0.0069	0.0069	n.s.	n.s.	0.0141	n.s.	**75**
**LEXF 2799**	0.0143	0.0082	0.0047	n.s.	n.s.	n.s.		0.0047	0.0253	0.0143	0.0047	0.0047	n.s.	0.0082	0.0082	0.0082	0.0027	0.0082	0.0143	0.0047	**22**
**LEXF 2824**	0.0082	n.s.	0.0027	0.0031	n.s.	0.0404	0.0047		0.0227	0.0082	0.0016	0.0016	0.0019	n.s.	0.0035	0.0035	0.0184	0.0035	0.0082	0.0016	**25**

**LEXF 2848**	0.0455	0.0446	0.0143	0.0343	0.0405	0.0304	0.0253	0.0227		n.s.	0.0143	n.s.	0.0263	0.0494	0.0253	0.0253	n.s.	0.0448	0.0455	n.s.	**89**
**LEXF 2897**	0.0246	0.0189	0.0082	0.0148	n.s.	n.s.	0.0143	0.0082	n.s.		0.0042	n.s.	0.0103	0.0177	0.0101	0.0101	n.s.	0.0169	0.0246	n.s.	**76**
**LEXF 2918**	0.0082	0.0039	0.0027	0.0031	0.0018	0.0035	0.0047	0.0016	0.0143	0.0042		0.0017	0.0019	0.0027	n.s.	0.0047	0.0011	0.0029	0.0082	0.0031	**168**
**LEXF 2943**	n.s.	0.0039	0.0027	0.0031	0.0153	n.s.	0.0047	0.0016	n.s.	n.s.	0.0017		0.0019	0.0027	0.0045	0.0045	n.s.	0.0029	n.s.	n.s.	**86**
**LEXF 2957**	0.0103	0.0043	0.0019	0.017	n.s.	0.0332	n.s.	0.0019	0.0263	0.0103	0.0019	0.0019		0.0043	0.0043	0.0043	0.0008	0.0043	0.0103	0.0019	**22**
**LEXF 2964**	0.0177	n.s.	0.0047	0.0067	n.s.	0.0483	0.0082	n.s.	0.0494	0.0177	0.0027	0.0027	0.0043		0.0067	0.0067	n.s.	n.s.	0.0177	0.0027	**47.5**
**LEXF 2966**	0.0143	0.0084	0.0047	0.0067	0.0049	0.0069	0.0082	0.0035	0.0253	0.0101	n.s.	0.0045	0.0043	0.0067		0.0067	0.003	0.0069	0.0143	0.0067	**155**
**LEXF 2997**	0.0143	0.0084	0.0047	0.0067	0.0049	0.0069	0.0082	0.0035	0.0253	0.0101	0.0047	0.0045	0.0043	0.0067	0.0067		0.003	0.0069	0.0143	0.0067	**149**
**LEXF 4010**	n.s.	n.s.	0.0016	0.0015	0.0407	n.s.	0.0027	0.0184	n.s.	n.s.	0.0011	n.s.	0.0008	n.s.	0.003	0.003		n.s.	n.s.	n.s.	**54.5**
**LEXF 4052**	0.0169	0.0084	0.0047	0.0067	n.s.	n.s.	0.0082	0.0035	0.0448	0.0169	0.0029	0.0029	0.0043	n.s.	0.0069	0.0069	n.s.		0.0169	0.0308	**53**
**LEXF 4096**	0.0253	0.0189	0.0082	0.0148	0.0136	0.0141	0.0143	0.0082	0.0455	0.0246	0.0082	n.s.	0.0103	0.0177	0.0143	0.0143	n.s.	0.0169		n.s.	**91**
**LEXF 4128**	n.s.	0.0039	0.0027	0.0031	0.0153	n.s.	0.0047	0.0016	n.s.	n.s.	0.0031	n.s.	0.0019	0.0027	0.0067	0.0067	n.s.	0.0308	n.s.		**105**

**median OS**	**79**	**44**	**24**	**24**	**33**	**75**	**22**	**25**	**89**	**76**	**168**	**86**	**22**	**47.5**	**155**	**149**	**54.5**	**53**	**91**	**105**	**64.75**

Abbreviations: n.s., not significant

© 2020 by the authors. Licensee MDPI, Basel, Switzerland. This article is an open access article distributed under the terms and conditions of the Creative Commons Attribution (CC BY) license (http://creativecommons.org/licenses/by/4.0/).
